# Implementation of REDCap mobile app in an oral HIV clinical study

**DOI:** 10.1186/s12889-024-17837-y

**Published:** 2024-02-28

**Authors:** Ana Lucia Seminario, Ashley E. Karczewski, Sara Stanley, Javier Valencia Huamani, Juan José Montenegro, Karla Tafur, Ana Bautista Julca, Frederick L. Altice

**Affiliations:** 1 Timothy A. DeRouen Center for Global Oral Health, University of Washington School of Dentistry, 1959 NE Pacific St B-307, Seattle, WA 98115 USA; 2 Universidad Peruana Cayetano Heredia, Lima, Peru; 3 Asociación Civil Impacta Salud y Educación, Lima, Peru; 4 Centro de Investigaciones Tecnológicas, Biomédicas y Medioambientales, Universidad Nacional Mayor de San Marcos, Lima, Peru; 5 Facultad de Ciencias de La Salud, Universidad Científica Del Sur, Lima, Peru; 6 Servicio de Medicina de Enfermedades Infecciosas y Tropicales, Hospital Nacional Dos de Mayo, Lima, Peru; 7 AIDS Program, Department of Internal Medicine, Section of Infectious Diseases, Yale University School of Medicine, New Haven, Connecticut USA; 8 Department of Epidemiology of Microbial Diseases, Yale School of Public Health, New Haven, Connecticut USA

**Keywords:** Mobile app, HIV, Oral health

## Abstract

**Background:**

In Peru, HIV cases are highly concentrated among men who have sex with men (MSM). Despite the availability of anti-retroviral therapy, people living with HIV (PWH) have higher levels of oral diseases. Alcohol use disorder (AUD) is significantly present among PWH. Our overarching goal was to generate foundational evidence on the association of AUD and oral health in MSM with HIV and enhance research capacity for future intersectional research on AUD, oral health and HIV. Our specific aim was to implement an on-site electronic data collection system through the use of a REDCap Mobile App in a low-middle income country (LMIC) setting.

**Methods:**

Five validated surveys were utilized to gather data on demographics, medical history, HIV status, alcohol use, HIV stigma, perceived oral health status, and dietary supplement use. These surveys were developed in REDCap and deployed with the REDCap Mobile App, which was installed on ten iPads across two medical HIV clinics in Lima, Peru. REDCap app as well as the protocol for data collection were calibrated with feedback from trial participants and clinical research staff to improve clinical efficiency and participant experience.

**Results:**

The mean age of participants (*n* = 398) was 35.94 ± 9.13y, of which 98.5% identified as male, and 85.7% identified as homosexual. 78.1% of participants binge drank, and 12.3% reported being heavy drinkers. After pilot testing, significant modifications to the structure and layout of the surveys were performed to improve efficiency and flow. The app was successfully deployed to replace cumbersome paper records and collected data was directly stored in a REDCap database.

**Conclusions:**

The REDCap Mobile App was successfully used due to its ability to: (a) capture and store data offline, (b) timely translate between multiple languages on the mobile app interface, and (c) provide user-friendly interface with low associated costs and ample support.

**Trial registration:**

1R56DE029639-01.

## Introduction

In Peru, HIV cases are highly concentrated among men who have sex with men (MSM). While HIV prevalence in the overall Peruvian adult population is estimated at 0.4%, the prevalence of HIV in the Peruvian MSM population is 10% [1]. This difference is even more evident in Lima, the capital of Peru where the prevalence of HIV is 100-fold higher in MSM relative to the general population (22.3% vs 0.2%) [2]. Due to strong social stigma against homosexuality, HIV rates among MSM is likely to be higher than currently reported, as reflected in the high percentage of MSM in Latin cultures who self-identify as heterosexual even when they have sex with men [3]. People living with HIV (PWH) have significant comorbidities [4, 5]. Despite the availability of anti-retroviral therapy (ART), PWH have higher levels of oral diseases such as dental caries and periodontal disease compared to non-HIV infected individuals [4, 5]. Yet, studies assessing the oral health of MSM with HIV are very limited [6].

Additionally, alcohol use disorders (AUD) are significantly present among PWH [7]. In Peru, there is an increase in heavy episodic drinking (i.e., binge drinking), and 14.0% of Peruvian males have an AUD [7]. AUD is thought to be more prevalent in PWH, especially MSM, due to alcohol’s disinhibitory effects that can lead to unsafe sex [8, 9]. AUD in PWH has also been observed to have a negative influence on ART adherence and subsequent HIV management [8, 9]. Although AUD has been associated with non-communicable diseases (NCDs), there is sparse research into the extent to which AUD severity mitigates these NCDs, including oral diseases [10, 11]. Alcohol consumption is noteworthy in Peru and has been shown to be a common risk factor for NCDs [7, 12]. AUDs are also associated with both poor general health and HIV-related outcomes. Due to the prevalence of HIV and AUD in MSM in Lima, Peru, this was an ideal location to conduct our behavioral and attitude assessment study examining the intersectionality of HIV, oral health, and AUD.

Large-scale studies require efficient, high-quality data to maintain human subject safety and to accurately track collected data. However, in low-and middle-income countries (LMICs), high-quality data collection, data utilization, and the sharing of aggregate data can be challenging due to a lack of reliable infrastructure such as Wi-Fi, electricity, and technology [13–15]. REDCap is an open-source platform that provides free, secure, HIPAA compliant web-based data capture, management, analysis and export [16]. REDCap is ubiquitous in research because it is browser-based and user friendly, low-no cost, and has an ample amount of free support and tutorials available online. While it has been successfully used in LMIC settings, even among those with limited connectivity [17, 18], its usage in oral health studies in developing countries is scarce. In a 2015 update, REDCap launched the REDCap Mobile App, to allow for offline data capture. In areas with low internet connectivity, REDCap Mobile App is a feasible option for data capture and management [1]. In this study, we implemented the use of the REDCap Mobile App in two medical clinics known for their vast experience in HIV research in Peru: IMPACTA San Miguel Clinical Research Site (IMPACTA), and Centro de Investigaciones Tecnologías, Biomédicas y Medioambientales (CITBM) [19–21]. Specifically, we worked with IMPACTA and CITBM clinic tablets, allowing our study team to capture data with intermittent and unreliable internet access. The goal of this study was to demonstrate feasibility for electronic data collection that integrates oral health within HIV research, in Lima, Peru.

## Methods

### Study population

The Institutional Review Boards of Washington University (STUDY00014519) as well as the collaborating Peruvian institutions ethical review committees of CITBM and IMPACTA approved this study. Informed consent was obtained from all subjects prior to participation. Participants were enrolled at two clinics that belong to the Peruvian Clinical Trial Unit (CTU). CTU Peru has a long-standing relationship and funding from NIH and funds the *HIV Prevention Trials Network*, *HIV Vaccine Trials Network*, *Microbicides Trials Network*, *AIDS Clinical Trials Group*, and *International Network for Strategic Initiatives in Global HIV Trials*. CTU has the infrastructure and training to conduct clinical trial research projects that meet Peruvian and US regulatory standards. Within the CTU, two clinics located in Lima served as our research sites, IMPACTA and CITBM. Both sites have demonstrated the ability to recruit and enroll a large number of MSM with and without HIV and AUD [19–21].

Our study population consisted of MSM with HIV who were being treated with antiretroviral therapy (ART) in Lima, Peru. Four-hundred participants between two urban clinic sites in Lima consented to participate in the study. Participant selection was determined by the following inclusion criteria: (1) at least 18 years of age, (2) have a known positive HIV diagnosis, (3) agree with the procedures of the study, and (4) be willing and able to give informed consent. Exclusion criteria included any medical, psychiatric, or other conditions that, in the investigator’s judgment, would interfere with or be a contraindication to the protocol procedures.

### Instruments used for data collection

The overarching goals of our research were to generate foundational evidence on the association of AUD and oral health in MSM with HIV and enhance research capacity for future intersectional research on oral health and HIV. To capture information from these participants and meet project objectives, five surveys were deployed. The first two surveys, *CASI_Yale_*1 (Survey 1) and *CASI_Yale_2* (Survey 2) were English-language surveys that were developed, validated, and translated into Spanish by a certified translator. *CASI_Yale_1* consisted of 36 questions that captured demographics (i.e., age, sex, sexuality, education, income, and profession), HIV experience and treatment adherence, perceptions of the study (i.e., preferences in clinic and study staff, expected compensation). *CASI_Yale_2* consisted of 26 questions capturing data such as personal health perceptions, alcohol and tobacco use, sexual practices, and food/housing security. Binge drinking was defined as 5 or more drinks within approximately two hours at least 1 time in the past month. Heavy drinking was defined as consuming more than 4 drinks on any day, or 14 drinks per week [22]. The third survey, *Oral Health* captured oral health perceptions and experiences from 8 questions asking about their dental/oral history and 15 questions with images of different oral diseases and asking if they had experienced them. The *Oral Health* survey was based on a validated survey of self-reported measures of periodontitis and oral health [23–25]. The fourth survey, *Vitamin D* had one question asking if the participant takes any vitamin supplements, and if so, which ones and how frequently. The final survey, *HIV Stigma*, was an adaptation of the Berger HIV Stigma scale-10 questions about the participants experience with stigma while living with HIV [26]. Across all surveys, a variety of question-and-answer formats were used. The types of formats included: single choice answer bubbles (i.e., multiple choice, agreement scale, and true/false), multiple checkbox responses, open-end free-text responses, and an “Other” answer choice with a prompted fill in the blank.

### Development of REDCap mobile app

**Fig. 1:**
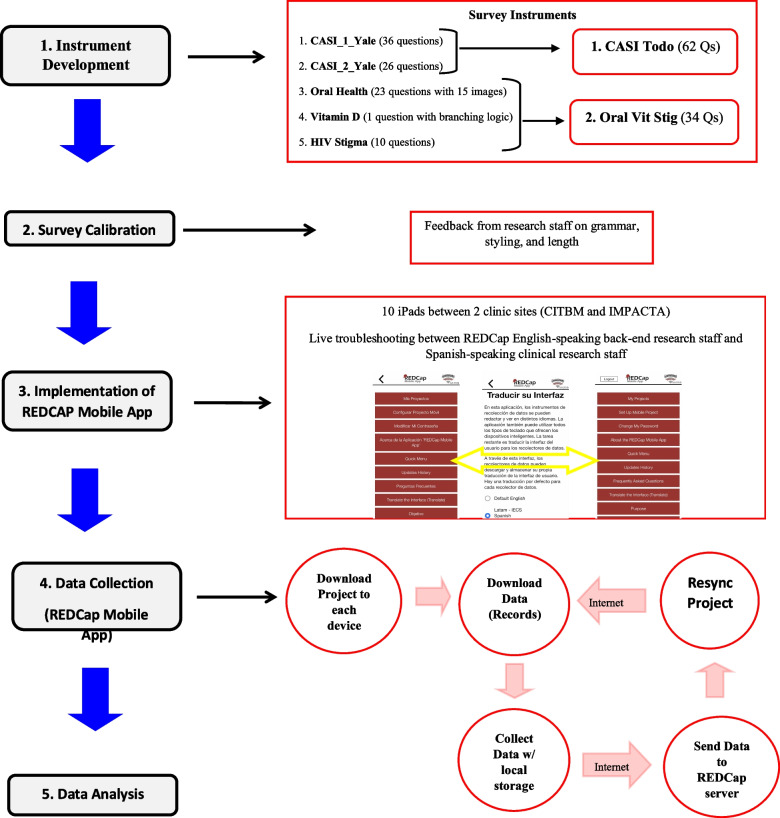
R56 VITA development and implementation

**Fig. 2:**
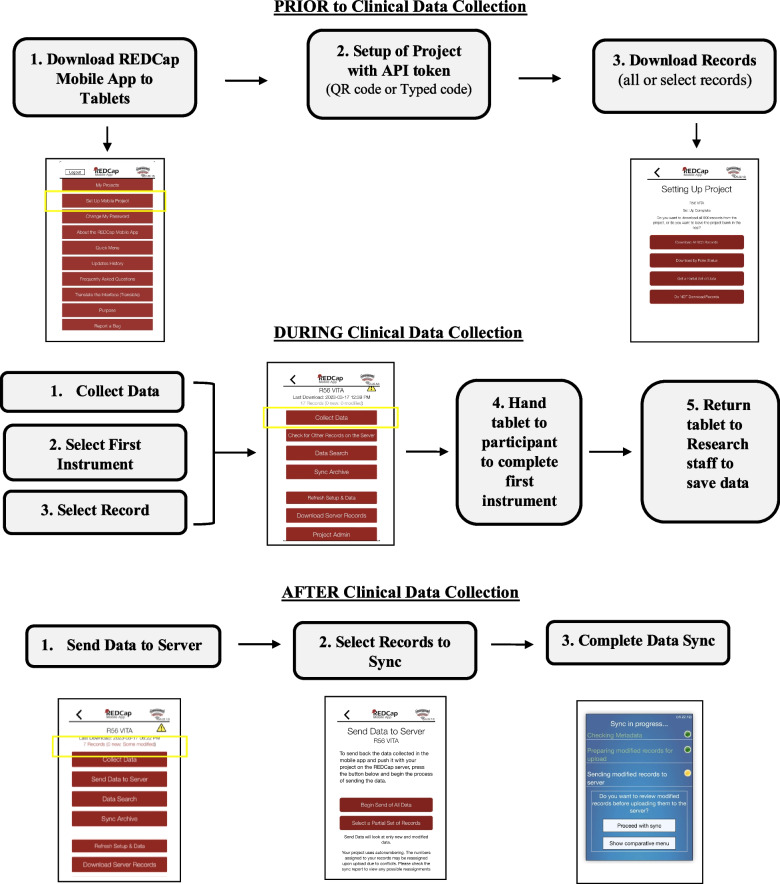
Clinical flow with REDCap mobile app

Study data were collected and managed using REDCap electronic data capture tools hosted at the University of Washington. REDCap is a secure, web-based software platform designed to support data capture for research studies, providing (1) an intuitive interface for validated data capture; (2) audit trails for tracking data manipulation and export procedures; (3) automated export procedures for seamless data downloads to common statistical packages; and (4) procedures for data integration and interoperability with external sources [16]. Originally, surveys were intended to be taken online in one of the multiple REDCap survey accessibilities such as QR code or email link. However, due to the unreliability of internet access at the site locations, REDCap Mobile App was deployed (Fig. 1). Eight iPads and two Android tablets were distributed amongst research staff at IMPACTA and CITBM for data collection. An application programming interface (API) token was enabled on the REDCap server and REDCap Mobile app was subsequently installed on all devices. Project data and empty records were uploaded on each tablet. Study staff were trained in collecting data from participants with iPads. Clinical flow was as follows: study staff confirmed the participants record number and gave the iPad to the participant to fill out the first survey; and then the participant was instructed to return the iPad to the staff after completing each survey so it could be properly saved, and the next survey could be queued up for completion. Following each day of data collection, study staff were to upload the participant data from that day by establishing a secure internet connection and syncing the project to the REDCap server (Fig. 2). If internet connection was unable to be secured, an emergency data dump was performed to save the data locally to the device for when a secure internet connection could be established.

### Survey calibration

Upon completion of designing the surveys in REDCap, the Peruvian medical research team reviewed each survey and provided feedback to the REDCap developer. After the initial revisions, the Peruvian clinical research staff were asked to complete the surveys on the iPads and provide further revisions and recommendations, for which they were compensated for their time. The final phase of survey calibration and revision involved enlisting a small cohort of participants who met the study inclusion criteria and were compensated for their time. They were asked to complete the surveys and provide further feedback on the surveys and REDCap Mobile App process. At this time, clinical research staff conducted a pilot study by collecting and then syncing data to ensure all data was accurate and able to be easily analyzed by back-end research staff. Feedback was communicated with the REDCap programmer, and subsequent changes were made until the surveys were grammatically, culturally, and procedurally correct. Once the surveys, instruments, and protocol were optimized, the clinical research team began gathering data at a rapid pace (Fig. 1).

## Results

### Study population

Data was collected from 398 individuals (99.5% of participants) (Table 1). The mean age of participants was 35.94 ± 9.13y, of which 98.5% identified as male, and 85.7% identified as homosexual. Most participants had higher education (post-secondary education) (67.8%), lived with a relative (45.7%) and rarely (35.7%) or never (33.2%%) experienced financial instability in the last year. Binge drinking was present among 78.1% of participants while 12.3 reported being heavy drinkers (Table 1).

**Table 1:** Characteristics of study population (*N* = 398)

**Variables (*N* = 398)**	**N (%)**
**Age (mean (SD), median (IQR)**	
Mean (SD)	35.94 (9.13)
Median (IQR)	35 (29–42)
**Education**	
1 Primary	5 (1.3%)
2 Secondary	123 (30.9%)
3 Superior	270 (67.8%)
**Sex**	
Male	392 (98.5%)
Female	1 (0.3%)
**Living status**	
Alone	101 (25.4%)
Live with a partner	86 (21.6%)
Live with a relative	182 (45.7%)
Other	29 (7.3%)
**Sexual orientation**	
Homosexual	341 (85.7%)
Bisexual	36 (9.1%)
Heterosexual	11 (2.8%)
Others	10 (2.5%)
**Financial instability in last year**	
Never	132 (33.2%)
Rarely	142 (35.7%)
Monthly/Daily/Weekly	110 (27.6%)
**Risk of AUD**	
No risk (AUDIT score = 0)	20 (5%)
Low risk (1–7)	212 (53.3%)
Harmful (8–14)	125 (31.4%)
AUD (> 15)	41 (10.3%)
**Binge Drink**	
No	87 (21.9%)
Yes	311 (78.1%)
**Heavy Drink**	
No	349 (87.7%)
Yes	49 (12.3%)

### Feedback on the implementation of the REDCap tool

During the initial development of the surveys and instruments for this study, over 100 h of development, training and background were required to make the research instruments user-friendly on both the back end and during data collection. Most REDCap survey features were not able to be enabled when implemented within the mobile app, such as survey pagination, branching logic, and question formatting. Survey pagination and question formatting is usually an important aspect of survey design, in order to make clear the flow and separation of topics to the user. Branching logic is another very helpful feature in designing REDCap surveys as it allows for exposure of a specific question based on the answer to a previous question. While branching logic is able to be implemented in the REDCap Mobile App, excessive use can significantly slows loading and downloading times during data capture. All of these features required manual tinkering and optimization from a traditional REDCap survey in order to fit the REDCap mobile app for this particular study. Embedded photos needed to be rescaled in proportion to the mobile app scaling, and question structure had to be reorganized for better viewing on a smaller screen. Certain surveys such as the *Oral Health* and the *HIV Stigma* surveys had to be restructured from matrix-based responses to simple checkbox or radio button answers and each question listed separately.

After the development of the REDCap surveys and instruments, the Peruvian medical research team spent two days providing feedback to the developer. The main feedback during this period improved the logical flow of the surveys and clarified questions and response choices to be clearer for participants and the Peruvian data collectors. The main feedback item from the clinical research staff was the tediousness of the participant needing to communicate with the research assistant between each of the 5 surveys. Thus, the 5 survey instruments were condensed into two larger surveys*, Casi Todo* (*CASI_Yale_1* and *CASI_Yale_2* surveys) and *Oral Vit Stig* (*Oral Health, Vitamin D,* and *HIV Stigma* surveys). The individual surveys were still demarcated within the two condensed instruments to allow for selective data analysis. The small pilot cohort provided further feedback concerning vocabulary used in the questions and suggested incorporating fun images to engage the participants and keep attention. During the clinical implementation, branching logic was modified to reduce the number of popups within a question, and to be more intuitive and efficient. The amount of time to complete both condensed surveys was approximately 40–50 min.

### REDCap mobile app limitations

There were several limitations to implementing the REDCap Mobile App for this study. First, while the app is an excellent tool to use in internet-limited areas, the app itself is cumbersome and procedurally intensive. There are multiple steps to download the saved data from the device, which was tedious to follow for clinic staff. Because of this complexity, a dedicated research staff member familiar with REDCap and the mobile app was required to be on site for the duration of the study to troubleshoot and ensure all data was entered and synced correctly.

## Discussion

The main objective of this descriptive study was to evaluate and describe the feasibility of integrating oral health research into HIV-related research among HIV positive Peruvian MSM using electronic data collection in a LMIC with unreliable internet access. Computer assisted self-interviewing surveys regarding HIV status and stigma, oral health, alcohol use were adapted to the REDCap Mobile App to replace cumbersome paper records without the need for technological infrastructure. It was hypothesized that utilizing the REDCap Mobile App for high-quality data collection and management would be feasible and efficient in collecting large amounts of data from a large cohort of patients. Use of the REDCap Mobile App in LMICs could expand the capacity of oral health research, integrating oral health records with medical records.

This study utilized the REDCap Mobile App as a reliable data collection tool in a LMIC with intermittent internet access. The app was successfully deployed to replace cumbersome paper records while maintaining independence from internet-dependent data collection. The most important and relevant features of REDCap in conducting global oral health research, as demonstrated in this study were: (1) Ability to capture and store data offline, (2) ability to quickly translate between multiple languages on the mobile app interface, and (3) user friendly interface with low associated costs and ample support. The ability to capture data offline is most important due to unreliable internet and high costs associated with purchasing reliable infrastructure, especially in LMICs. Currently, most data collection in LMICs is done with paper records due to the lack of reliable infrastructure and devices [13–15]. However, REDCap Mobile App avoid many of the hassles and pitfalls of paper records. In particular, the ability to use photos in the survey instruments is relevant to global oral health research, as often gold-standard means of diagnosis such as radiographic images cannot be used to diagnose dental caries and periodontitis in resource limited settings. Thus, visual clinical examination is the main means of diagnosis. Photos are useful in determining oral disease burden by non-dental research staff, therefore being able to integrate oral health data into medical research. Likewise, there are many data points in oral health research, such as the 6 surfaces of each tooth, and electronic capture of this information greatly expedites data analysis. The mobile app can collect data offline and store it to the device until a reliable internet connection can be achieved. Once connected, the data is synced to the REDCap server and can be accessed from any computer’s internet browser. If there is only unreliable internet connection, emergency data dumps can be performed that save the data as an excel file, which must then be untangled by the data analyst. Data dumps are not ideal, but an important safety net in areas lacking infrastructure.

This project was initially developed with 5 Spanish-language surveys evaluating MSM with HIV demographics, HIV status and adherence, alcohol use, oral health, HIV stigma, and supplementation. The five initial surveys were combined into two demarcated surveys for better efficiency and reduced steps in the clinic. The implementation of clinic feedback for the development was greatly aided by the use of the REDCap Mobile App’s ability to toggle between dozens of languages quickly and easily. This feature was critical amongst a multinational team that spoke different languages. English-speaking team members were able to replicate the Spanish version of the app interface to help troubleshoot problems clinic staff were encountering, and vice versa. This rapid toggling improved communication amongst the team and increased the efficiency of the study deployment.

There were several limitations to this study. The nature of this research is a qualitative description of the implementation of an oral health data capture tool in a medical research setting with limited resources. Feedback from participants and research staff was not captured in any instruments and was mostly discussion based with limited quantitative feedback. Secondly, the implementation of REDCap Mobile App is best used in limited resource settings, as there are other mobile data capture tools that are more efficient and user friendly when internet access is not a major concern.

## Conclusions

The ubiquity of REDCap globally is mostly due in part to it being browser-based, its easy-to-use interface, and the number of free resources and support available for project development and implementation. Data from a large cohort in Lima, Peru was able to be successfully and efficiently collected, managed, and analyzed. Utilizing the REDCap Mobile app is a good bridge between paper records and electronic health record systems for research conducted in low resource settings.

## Data Availability

The intent of this study is to describe the use of the REDCap Mobile App and all demographic data mentioned is for setting and patient population context. The datasets used and/or analyzed during the current study are available from the corresponding author on reasonable request.

## Abbreviations

MSM: Men who have sex with men
PWH: People living with HIV
AUD: Alcohol use disorder
ART: Antiretroviral therapy
NCD: Non-communicable diseases
LMIC: Low-middle income country
NCRR: National Center for Research Resources
NIH: National Institutes of Health
IMPACTA: IMPACTA San Miguel Clinical Research Site
CITBM: Centro de Investigaciones Tecnologías, Biomédicas y Medioambientales
CTU: Clinical Trial Unit
